# Correction: What is your count? An observational study of lymph node counting in 2,028 colorectal cancer resections

**DOI:** 10.1371/journal.pone.0299858

**Published:** 2024-02-27

**Authors:** The PLOS ONE Staff

## Notice of Republication

This article was republished on February 14, 2024, to correct error in the authors’ affiliation that was introduced during the typesetting process. The publisher apologizes for this error. Please download this article again to view the correct version. The originally published, uncorrected article and the republished, corrected articles are provided here for reference.

## Supporting information

S1 File Originally published, uncorrected article. (PDF)

S2 File Republished, corrected article. (PDF)
