# Supplementary material for: What is your count? An observational study of lymph node counting in 2,028 colorectal cancer resections
Source: PLoS One. 2024 Feb 8;19(2):e0295209. doi: 10.1371/journal.pone.0295209 (PMC10852306; doi:10.1371/journal.pone.0295209)
Supplement: S1 File — (DOCX) [file pone.0295209.s001.docx]

**Data Availability and Data File Description**

The minimal data set is included as supplemental materials; it contains the data necessary to build the figures and tables in the manuscript and supplemental materials.

The columns in the anonymized data files are as follows:

Acc_Year = accession year

T_STAGE = tumour stage; 0 = pTX, 1 = pT0, 2 = pTis, 3 = pT1, 4 = pT2, 5 = pT3, 6 = pT4, 7 = pT4a, 8 = pT4b

N_STAGE = nodal stage; 0 = pNX, 1 = pN0, 2 = pT1, 3 = pN1a, 4 = pN1b, 5 = pN1c, 6 = pN2, 7 = pN2a, 8 = pN2b

Y_VAR = y modifier status; 1 = absent, 2 = present

LNcount = lymph node count

LNpos = positive lymph node count

TDpos = tumour deposits; 0 = tumour deposits absent, 1 = tumour deposits present

TDcount = tumour deposits count

PATHOLOGIST = anonymous pathologist identifier

The complete data set (n=2,543) is contained in the file: “S2”

The trimmed data set (n=2,028) is contained in the file: “S3”
