# Supplementary figures and images for: What is your count? An observational study of lymph node counting in 2,028 colorectal cancer resections
Source: PLoS One. 2024 Feb 8;19(2):e0295209. doi: 10.1371/journal.pone.0295209 (PMC10852306; doi:10.1371/journal.pone.0295209)

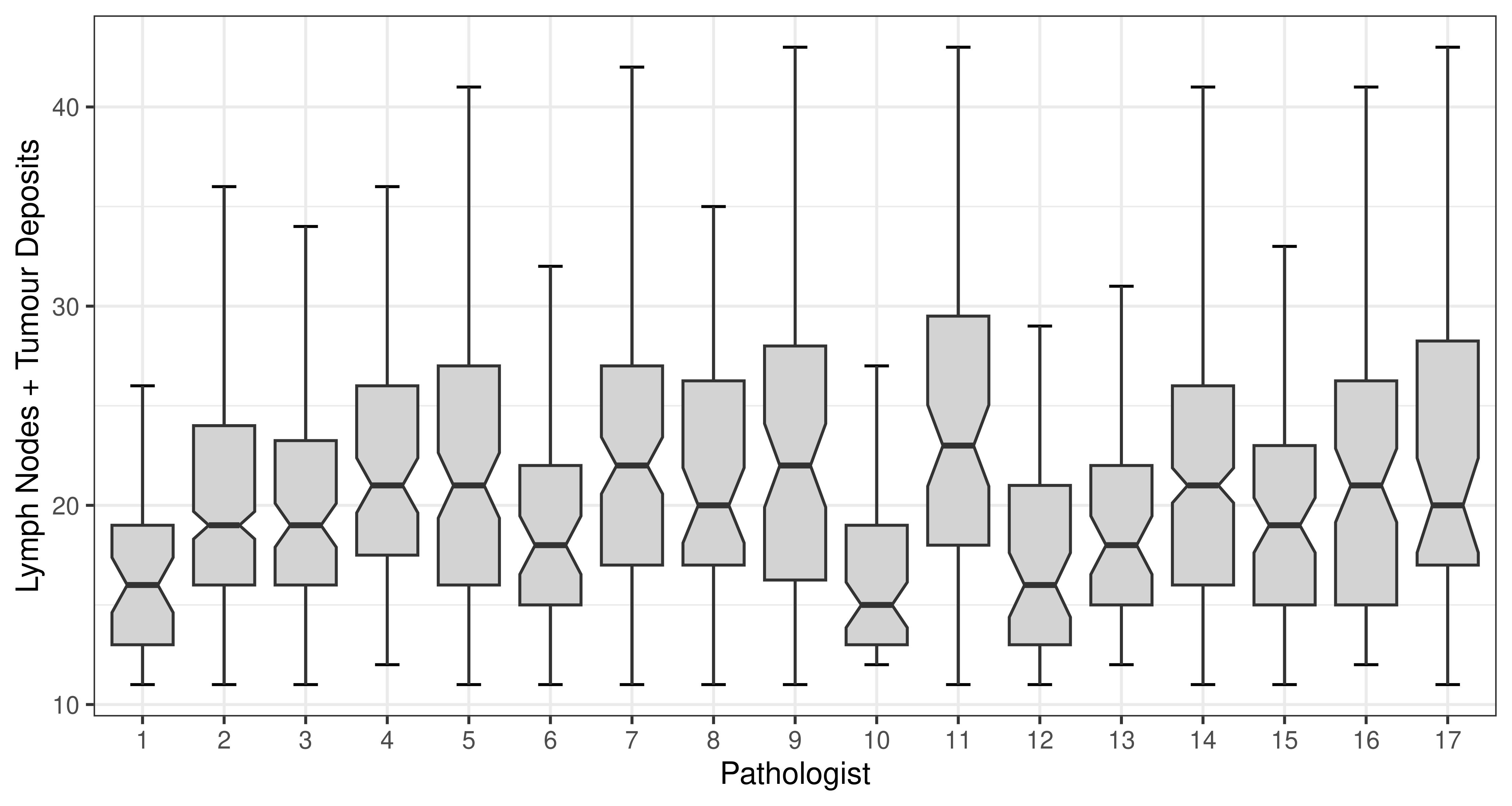

Supplement: S1 Fig — (TIF) [file pone.0295209.s005.tif]

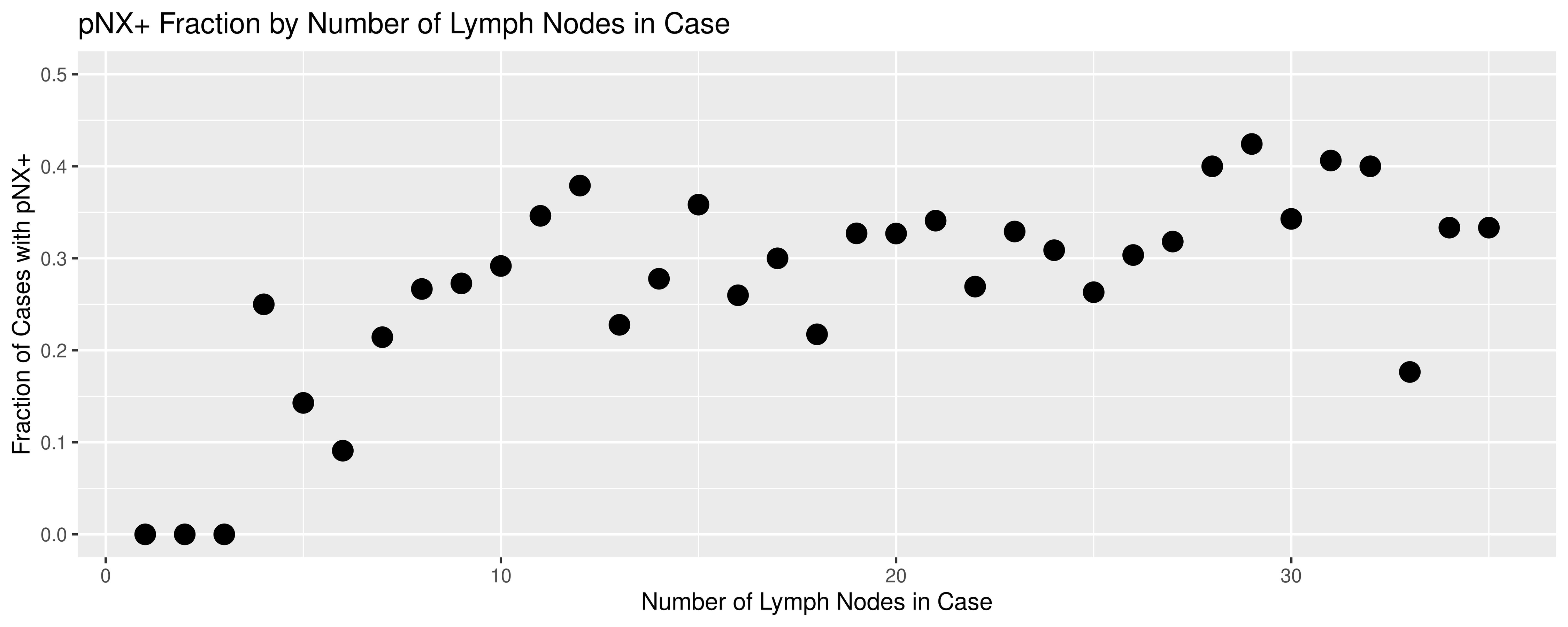

Supplement: S2 Fig — (ZIP) [file pone.0295209.s006.zip › Figure_S2a__LN_Metastasis_Rate_vs_Lymph_Node_Count_PACE.tif]

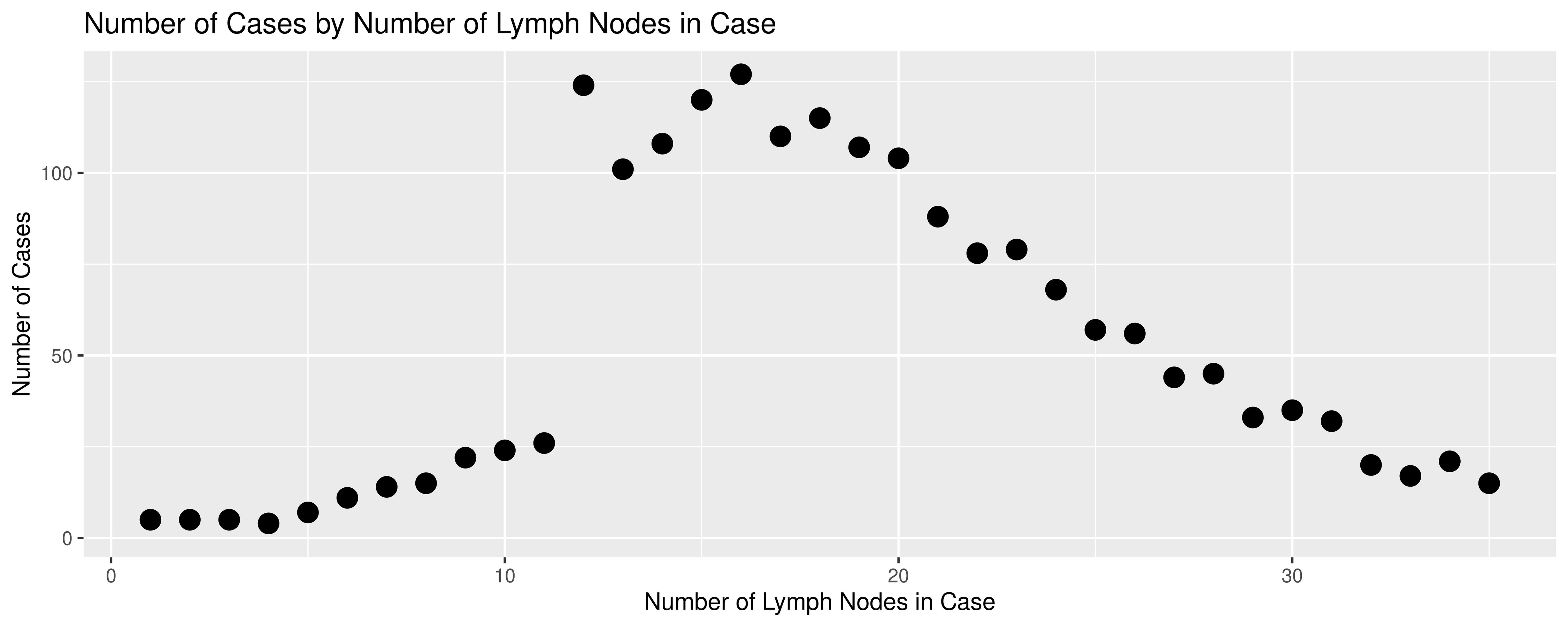

Supplement: S2 Fig — (ZIP) [file pone.0295209.s006.zip › Figure_S2b__Num_Cases_vs_Lymph_Node_Count_PACE.tif]
