# Supplementary material for: Correction: What is your count? An observational study of lymph node counting in 2,028 colorectal cancer resections
Source: PLoS One. 2024 Feb 27;19(2):e0299858. doi: 10.1371/journal.pone.0299858 (PMC10898766; doi:10.1371/journal.pone.0299858)
Supplement: S2 File — (PDF) [file pone.0299858.s002.pdf]

PLOS ONE

RESEARCH ARTICLE

What is your count? An observational study of lymph node counting in 2,028 colorectal cancer resections

Shivali Srivastava[1], Ipshita Kak[1], Pierre Major[2], Michael Bonert[1]*

1 Pathology and Molecular Medicine, McMaster University, Hamilton, ON, Canada, **2** Medical Oncology, McMaster University, Hamilton, ON, Canada

* bonertm@mcmaster.ca

Abstract

Background

Lymph node status and lymph node count (LNC) are predictors of colorectal cancer outcome. Under-sampling of lymph nodes may lead to clinically relevant stage migration.

Methods

Colorectal cancer (CRC) cases with a synoptic report, accessioned 2012–2020 at a regional laboratory, were extracted and retrospectively studied. LNC, positive lymph node count (PLNC), tumour deposits present (TDpos), and 'y' (staging) prefix (YS) were retrieved and tabulated by pathologist using custom software. Statistical analyses were done with R.

Data and results

The cohort had 2,543 CRC resections. Seventeen pathologists interpreted >50 cases (range: 56–356) each and collectively saw 2,074. After cases with unavailable data were purged, 2,028 cases remained with 43,996 lymph nodes, of which 2,637/43,996 were positive. 368 cases had a 'y' prefix, and 379 had TDpos. The 17 pathologists' median LNC/case was 19.0 (range: 14.0–24.0), and the mean PLNC per case was 1.4 (range: 1.0–2.0). Kruskal-Wallis rank sum tests showed there were differences in LNC (p<0.001) among pathologists; however, PLNC did not show this association (p = 0.2917). T-tests showed that mean LNC (p<0.001) and PLNC (p<0.035) differed between YS. 138 of 2,028 cases had less than the 12 LNC target. Logistic regression revealed a strong association between meeting the LNC target and pathologist (p<0.001) but TDpos was non-predictive (p = 0.4736).

Conclusions

Positive lymph node call rate has a good consistency in the laboratory; however, lymph node count varies significantly between pathologists. Standardized counting criteria are needed to improve uniformity and could be aided by synoptic reporting data.

Check for updates

OPEN ACCESS

Citation: Srivastava S, Kak I, Major P, Bonert M (2024) What is your count? An observational study of lymph node counting in 2,028 colorectal cancer resections. PLoS ONE 19(2): e0295209. https://doi.org/10.1371/journal.pone.0295209

Editor: Antonino Maniaci, University of Enna Kore: Universita degli Studi di Enna 'Kore', ITALY

Received: July 20, 2023

Accepted: November 15, 2023

Published: February 8, 2024

Copyright: © 2024 Srivastava et al. This is an open access article distributed under the terms of the Creative Commons Attribution License, which permits unrestricted use, distribution, and reproduction in any medium, provided the original author and source are credited.

Data Availability Statement: The minimal data set is included as supplemental materials; it contains the data necessary to build the figures and tables in the paper and supplemental materials.

Funding: The author(s) received no specific funding for this work.

Competing interests: The authors have disclosed that they have no significant relationships with, or financial interest in, any commercial companies pertaining to this article. The corresponding author (MB) retains the copyright on the computer code that was written outside of his employment

relationship with McMaster University/St. Joseph's Healthcare Hamilton/Hamilton Regional Laboratory Medicine Program. The above does not in any way limit adherence to the PLOS ONE data availability policy, as found here: https://journals.plos.org/plosone/s/data-availability. There is no financial conflict of interest. There are no conflicts for the other authors.

Introduction

In malignancy, the pathologic assessment of lymph nodes determines cancer stage and prognosis, which in turn guides treatment. As such, lymph node assessments are a factor in the quality of care and are considered an important metric in quality assurance.

In 1993, Hermanek and Henson *et al*. published a hallmark paper on lymph node counting in cancers. It recommended that at least 12 lymph nodes be examined in colorectal cancer patients to accurately stage the disease [1]. In the subsequent years, the 12 lymph nodes count minimum (for colorectal resections) was adopted by the World Congress of Gastroenterology, National Cancer Institute (NCI) Consensus Conference on Colorectal Cancer and American Joint Committee on Cancer [2].

Studies have shown an association between the number of lymph nodes examined, the proportion of positive lymph nodes and the prognosis in cases of malignant colorectal resections [3–6]. One of these studies (Kelder *et al*. in 2009) retrospectively reviewed 2,281 patients with localized colon cancer; it revealed that the higher the number of lymph nodes examined, the greater the proportion of node positivity—thereby establishing a correlation that lymph node count can influence the positive lymph node rate [4].

Lymph node counts and sampling

Poorly sampled lymph nodes may be unrepresentative of the disease and lead to under-staging [4]. In a group of patients, this can lead to effects such as the *Will Rogers Phenomenon* (especially when considered in the context of treatment); when a patient cohort with poorly sampled lymph nodes is compared to a cohort with well sampled lymph nodes: a portion of the poorly sampled is under-staged and would undergo so-called "stage migration" if better sampled [7, 8]. The up-staged patients get more aggressive treatments (better tailored to the disease severity) and have an improved outcome [4–7].

Sources of variation

Variation in the lymph node count may be influenced by patient factors, tissue acquisition, laboratory processing, and the pathologist [9]. Prior studies on glass slides with pathologists have shown significant variation in the lymph node count [10, 11].

In Parkash *et al*. ten pathologists counted nodes on 15 slides on two separate occasions [11]. The results were sobering; they showed that there was no slide on which all pathologists agreed on both occasions. Sources of disagreement included: the smallest countable node size, counts of two closely related structures, and when the gross count conflicted with the microscopic impression.

Tumour deposits

The lymph node count in colorectal cancer is further complicated by "tumour deposits" for which criteria have changed significantly with AJCC editions. They are independent indicators of decreased patient survival and increased recurrence in numerous studies [12, 13]. Prior work has shown difficulty in reproducibly separating tumour deposits from lymph nodes [13]. A number of studies have suggested considering tumour deposits the same as lymph nodes when counting the number of locally metastatic disease foci, but the current scheme does not allow for this [12, 13]. Further, it is worth noting that different areas of the gastrointestinal tract have varied rules for tumour deposits; for example, in stomach, tumour deposits are counted as lymph node metastasis; the "N1c" category for tumour deposits does not exist in stomach cancer staging.

'y' TNM modifier and lymph node count

The "y" prefix in the TNM staging system indicates that the assessment follows therapy (e.g., neoadjuvant chemotherapy, radiation therapy, or both chemotherapy and radiation therapy). The "y" categorization is not an estimate of the tumour prior to multimodality therapy (i.e. before initiation of neoadjuvant therapy) [14]. A study by Chen *et al.* found that neoadjuvant therapy was associated with a lower lymph node count in colon cancer resection cases and that a low lymph node ratio (the proportion of positive lymph nodes to the total number of lymph nodes examined) was associated with worse outcomes in patients who received neoadjuvant therapy [15]. Another study by Nelson *et al.* found that neoadjuvant therapy was associated with a lower lymph node count in colon cancer resection cases compared to cases where neoadjuvant therapy was not given. However, the study also found that the effect of neoadjuvant therapy on the lymph node count varied depending on the type of therapy and the timing of the surgery [16].

Objective

The main objectives of this work are to assess the variation between pathologists in (1) the lymph node count, and (2) the positive lymph node count in colorectal cancer using observational data. Secondary objectives include assessing whether the 'y' staging modifier and/or the presence of tumour deposits have an impact on the lymph node counts.

Methods

Ethics board approval was obtained to retrieve all colorectal cancer resections for the time period 2012–2020 (Hamilton Integrated Research Ethics Board #4445). The study was done in accordance with national ethics guidelines and relevant regulations. Patient consent was not required by the ethics board, due to the study design. After data extraction the data set was anonymized.

Colorectal cancer cases with a synoptic report, accessioned January 1, 2012 to December 31, 2020 at a regional laboratory, were extracted on February 27, 2023. Lymph node count (LNC), number of positive lymph nodes (PLNC), tumour deposits present (TDpos), number of tumour deposits, and 'y' (staging) prefix (YS) were tabulated by pathologist using custom software. Statistical analyses were done with R (https://cran.r-project.org). Pathologist call rates were compared with funnel plot and control charts, using code available online (https://github.com/mbonert/cmpproviders). The box-whisker plot explanation was created from an example online (https://waterdata.usgs.gov/blog/boxplots/). Notched boxes were used on the box-whisker plots, where any two boxes with non-overlapped notches have approximately a 95% confidence interval or greater for the difference between the medians, as noted in the R documentation (https://www.rdocumentation.org/packages/grDevices/versions/3.6.2/topics/boxplot.stats); this would roughly correspond to $p \leq 0.05$.

Cancer synoptic reporting was instituted at a provincial government agency level (via Cancer Care Ontario) in the 2000s [17]. This work indirectly builds off that effort. As reports are centrally collected and the information submitted electronically in separate database fields, the individual data elements could be reconstructed from the local pathology reports.

The code that reconstructed the data navigated the complexity of the reporting environment. The time period included a change in the UICC/AJCC staging edition (from 7th to 8th edition). Cases were excluded from the analysis based on the following criteria: (1) the case was assessed by a pathologist that interpreted 50 or fewer cases, (2) the lymph node count was not available or less than one, (3) the positive lymph node count was less than zero, (4) the positive lymph node count exceeded the lymph node count.

Results

The cohort consisted of 2,543 colorectal cancer resections with a synoptic report in the studied time period. The 2,543 cases were read by 43 pathologists. Seventeen pathologists interpreted >50 cases (range: 56–356) each and collectively saw 2,074 cases.

After cases read by pathologists who encountered a low volume of colorectal cancer resections (< = 50 cases) and cases with unavailable data were purged, 2,028 cases remained. The forty-five cases with unavailable data that were purged were examined; 41 of these did not have a lymph node count, and five had a positive lymph node count greater than the lymph node count. The 2,028 cases that were further analyzed had a total of 43,996 lymph nodes of which 2,637 were positive for malignancy. The tumour stage and nodal stage were cross tabulated (see S1 Table in S3 Data) and tabulated by pathologist (see S2a and S2b Table in S3 Data). Fifteen reporting errors were found in the set of 2,028 cases, where lymph node count and nodal stage were inconsistent—details in S4 Table in S3 Data. The reported lymph node counts were presumed correct in these cases.

The group of 17 pathologists showed significant variation in the lymph node count, as demonstrated by the box and whisker plot (see Fig 1 and Table 1).

The mean of the mean PLNC per case (for all pathologists) was 1.4 and the mean PLNC per case had a modest range (1.0–2.0), as shown in Table 2.

The 'y' staging prefix was present in 368 of 2,028 cases and the lymph node count differed by the 'y' staging prefix (see Fig 2 and Table 3).

At least one tumour deposit (TDpos) was seen in 379 of 2,028 cases and the fraction of cases with tumour deposits by pathologist varied from 4.4% to 30.4% (see S2c Table in S3 Data). In logistic regression, the presence of tumour deposits (TDpos) was predicted by the pathologist in a univariate model ($p<0.001$).

Kruskal-Wallis rank sum tests showed that there were differences in lymph node count (LNC) ($p<0.001$) among pathologists; however, positive lymph node count (PLNC) did not show this association ($p = 0.2917$). T-tests showed that mean LNC ($p<0.001$) and PLNC ($p<0.035$) differed between YS.

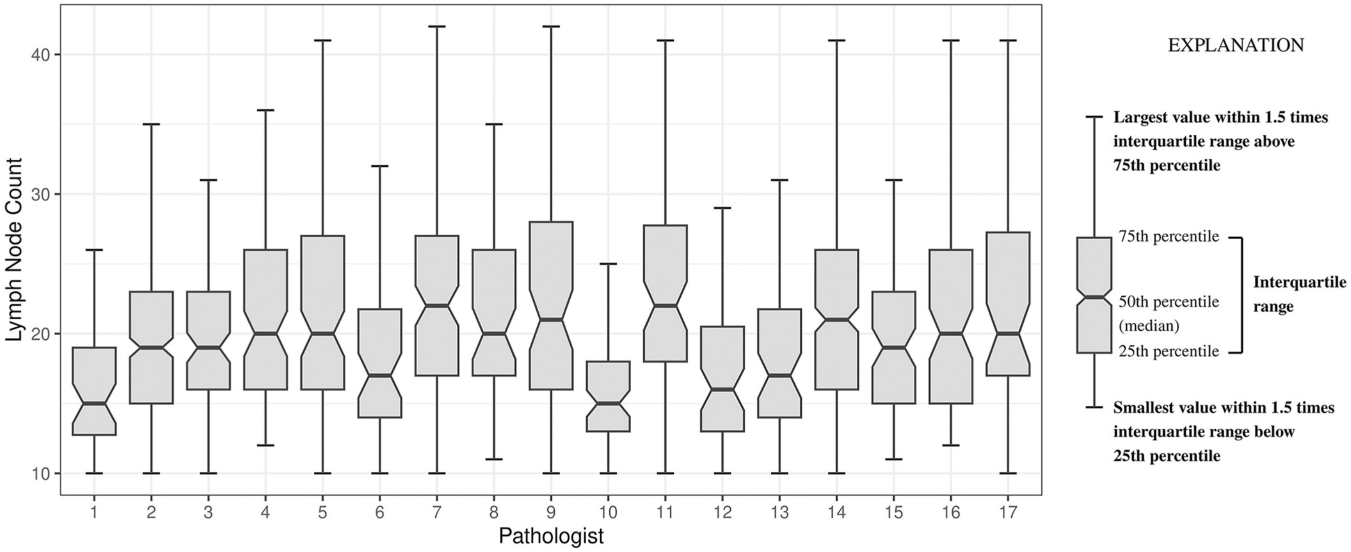

Fig 1. Box-whisker plot of lymph node count by pathologist (for pathologists interpreting greater than 50 cases). Any two boxes with non-overlapped notches have approximately a 95% confidence interval or greater for the difference between the medians; this would roughly correspond to $p<0.05$.

https://doi.org/10.1371/journal.pone.0295209.g001

Table 1. Lymph node count by pathologist.

Pathologist	Case Count	Mean LN	Median LN	Stdev LN	Max LN	Min LN
Pathologist 1	56	14.5	14	5.2	26	2
Pathologist 2	355	20.7	19	10.0	113	1
Pathologist 3	121	19.0	17	8.7	55	1
Pathologist 4	102	22.9	20	11.4	86	2
Pathologist 5	125	22.2	20	10.6	75	3
Pathologist 6	67	18.2	16	8.8	54	4
Pathologist 7	135	24.2	22	11.7	87	4
Pathologist 8	73	25.0	20	16.6	120	1
Pathologist 9	88	23.7	21.5	11.1	55	7
Pathologist 10	70	16.7	15	5.4	41	10
Pathologist 11	91	25.8	24	11.3	57	9
Pathologist 12	69	16.5	16	5.9	37	5
Pathologist 13	61	18.7	17	7.6	47	4
Pathologist 14	356	23.1	21	14.0	186	3
Pathologist 15	91	19.6	19	9.1	73	3
Pathologist 16	100	25.0	21	14.0	101	12
Pathologist 17	68	25.1	20	14.0	77	7
Mean	119.3	21.2	19.0	10.3	75.9	4.6
Median	91.0	22.2	20.0	10.6	73.0	4.0
Stdev	91.9	3.5	2.7	3.2	38.8	3.3
Max	356.0	25.8	24.0	16.6	186.0	12.0
Min	56.0	14.5	14.0	5.2	26.0	1.0

LN = Lymph node, Stdev = Standard deviation, Max = Maximum, Min = Minimum

https://doi.org/10.1371/journal.pone.0295209.t001

When the tumour deposit count (TDC) and lymph node count were added to form a lumped variable, Kruskal-Wallis rank sum tests again showed that there were differences in the count of the lumped variable ($p<0.001$) among pathologists; however, positive lymph node count (PLNC) again did not show this association ($p = 0.3549$). A box and whisker plot of the lumped variable is similar to the one for the lymph node count; compare Fig 1 to S1 Fig.

Twelve lymph node target

The lymph node count (LNC) target of 12 was missed in 138 of 2,028 cases (6.8%). Logistic regression revealed that there was a strong association between the LNC target of 12 lymph nodes and pathologist ($p<0.001$). In a multivariate model with the predictors pathologist and TDpos, pathologist remained significant ($p<0.001$) and TDpos was non-predictive ($p = 0.4736$).

Thirteen of 17 pathologists (~76%) had 12 or more lymph nodes in >90% of their cases (see Table 3). Five of 17 pathologists (~29%) had 12 or more lymph nodes in >95% of their cases.

Tumour stage

The effect of the T stage was also investigated. To avoid over-fitting, infrequent T stage categories were purged and yielded a data set with 1,988 cases. The most complex model to predict whether the LNC target was met included pathologist, YS, T stage and TDpos; in that model

Table 2. Positive lymph node count by pathologist.

Pathologist	Case Count	Mean Pos LN	Stdev Pos LN	Max Pos LN
Pathologist 1	56	1.3	2.9	13
Pathologist 2	355	1.1	2.6	22
Pathologist 3	121	1.0	1.9	14
Pathologist 4	102	2.0	4.8	31
Pathologist 5	125	1.2	2.6	13
Pathologist 6	67	1.3	2.8	14
Pathologist 7	135	1.3	2.4	17
Pathologist 8	73	1.7	4.1	25
Pathologist 9	88	1.5	3.5	21
Pathologist 10	70	1.9	3.0	14
Pathologist 11	91	1.1	2.6	14
Pathologist 12	69	1.3	2.7	10
Pathologist 13	61	1.1	2.5	16
Pathologist 14	356	1.1	3.0	45
Pathologist 15	91	1.6	2.7	11
Pathologist 16	100	1.4	3.0	15
Pathologist 17	68	1.5	4.7	31
Mean	119.3	1.4	3.0	19.2
Median	91.0	1.3	2.8	15.0
Stdev	91.9	0.3	0.8	9.2
Max	356.0	2.0	4.8	45.0
Min	56.0	1.0	1.9	10.0

Note: The median positive lymph node count is zero for all pathologists. The minimum positive lymph node count is zero for all pathologists.

https://doi.org/10.1371/journal.pone.0295209.t002

pathologist and YS were significant (both $p<0.001$), T stage was moderately significant ($p = 0.003$) and TDpos was not significant ($p = 0.7887$).

The lymph node positive rate was plotted against the lymph node count per case (see S2A Fig). The number of cases with a given lymph node count were also tabulated and plotted (see S2B Fig). A tabular form of these is found in supplemental materials (see S3 Table in S3 Data).

Discussion

A large number of factors are known to impact lymph node yields in colorectal cancer, including treatment/surgical factors (e.g. size of resection, targeted lymph node retrieval, neo-adjuvant treatment), patient factors (e.g. body habitus, patient age), pathology/pathologist factors (e.g., amount of tissue submitted, lymph node count at grossing, tissue processing, counting procedure/interpretative criteria) and tumour/biology factors (e.g. poor differentiation, depth of invasion and lymphovascular invasion [11]).

The work herein suggests that the pathology/pathologist factors are the dominant driver of variability, as the lymph node counts differ between providers in this observational study; however, the positive lymph node counts do not differ statistically. The underlying cause for the differences in the lymph node count is suspected to be a lack of standardization in pathology, and likely includes factors identified by Parkash *et al.* [11]. As suggested by Sherbeck *et al.*, there is a need for a consensus on assessing and reporting lymph node counts [10]. If too

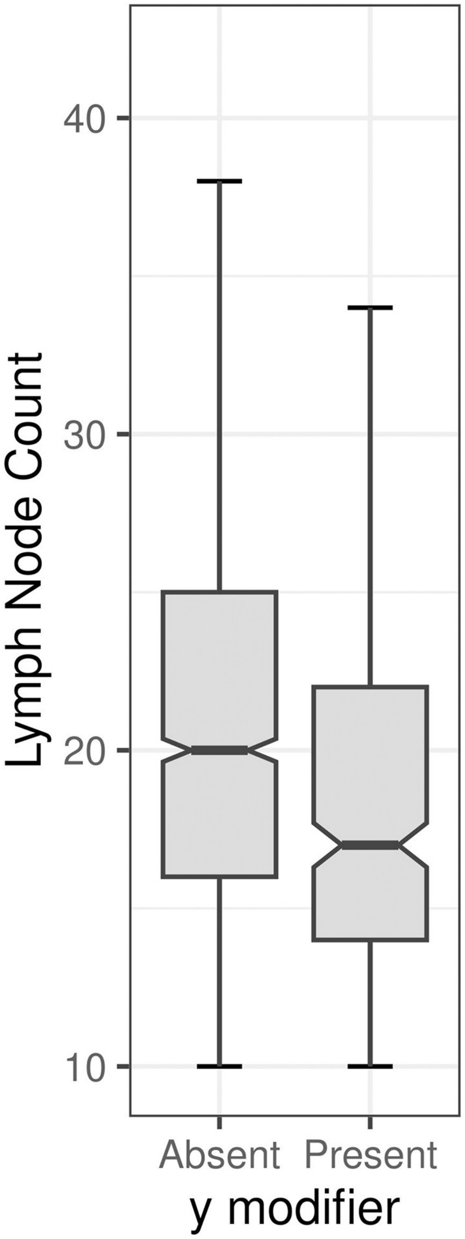

Fig 2. Lymph node count by 'y' TNM staging modifier status. The non-overlap of the notches is in keeping with a significant statistical difference ($p<0.05$) between the two groups, as also found with a T-test.

https://doi.org/10.1371/journal.pone.0295209.g002

few lymph nodes are retrieved, prior work has suggested methods to increase the count [18–20].

Beyond a lacking consensus on 'how to count', we suspect that a fundamental issue is a lack of consistent (routine) assessment of the variability in the lymph node count within a quality assurance context.

Our prior work examining diagnostic consistency suggests that pathologists have stable diagnostic rates [21] and that those rates with individualized feedback are modifiable, i.e. the diagnostic consistency could be improved via next generation quality [22].

Table 3. y modifier by pathologist & > = 12 lymph nodes.

Pathologist	Count	y present	y rate	Not 12 LN	Not 12 LN rate
Pathologist 1	56	5	0.089	13	0.232
Pathologist 2	355	97	0.273	16	0.045
Pathologist 3	121	32	0.264	13	0.107
Pathologist 4	102	10	0.098	2	0.020
Pathologist 5	125	8	0.064	11	0.088
Pathologist 6	67	22	0.328	12	0.179
Pathologist 7	135	8	0.059	7	0.052
Pathologist 8	73	11	0.151	5	0.068
Pathologist 9	88	11	0.125	6	0.068
Pathologist 10	70	6	0.086	1	0.014
Pathologist 11	91	14	0.154	4	0.044
Pathologist 12	69	9	0.130	11	0.159
Pathologist 13	61	12	0.197	4	0.066
Pathologist 14	356	93	0.261	21	0.059
Pathologist 15	91	18	0.198	6	0.066
Pathologist 16	100	9	0.090	0	0.000
Pathologist 17	68	3	0.044	6	0.088
Sum	2028	368	0.181	138	0.068

https://doi.org/10.1371/journal.pone.0295209.t003

The introduction and adoption of digital pathology may open up several possibilities; a complex algorithm could be developed to count lymph nodes that makes use of (1) the lymph node count at grossing, (2) the distance between possible lymph nodes, (3) the size of the possible lymph node, (4) the contour of the possible lymph node, (5) presence of tumour within the possible lymph node, and (6) the distance/relation of the possible lymph node to blood vessels.

‘y’ TNM modifier and lymph node count

The findings of this study are in keeping with prior work [23] that demonstrated that lymph node counts are lower in cases with a ‘y’ modifier.

Tumour deposits and lymph node counts

In this study, tumour deposits were identified in 379 of 2,028 cases (18.7%), and the presence of tumour deposits was predicted by the pathologist in a univariate model ($p<0.001$).

The higher p value ($p = 0.3549$) obtained from the analysis on the lumped variable (PLNC +TDC) versus PLNC alone ($p = 0.2917$) suggests some variation arises due to the determination: positive lymph node *versus* tumour deposit. These findings are in keeping with prior work showing variation in designating and differentiating tumour deposits from positive lymph nodes [24].

Positive lymph node count versus total lymph node counting

The difference in the statistical findings for lymph node count and positive lymph node count may be explained by several factors. The sample size is likely a major factor—positive lymph nodes are much less numerous. Morphology is suspected to be a factor, as “round” lymph nodes are more likely to be malignant [25]. Inter-rater agreement may be higher for round

lymph nodes without significant inflections of the surface contour, as they are less likely to mimic two lymph nodes in a plane-of-section.

Twelve lymph node target

The lymph node count (LNC) target of 12 was confirmed as a rational target; the lymph node positive rate above 12 lymph nodes was essentially independent of the lymph node count. Below 12 lymph nodes cases were likely under-staged.

A percentage of cases achieving the 12 lymph node count target would be useful as a quality benchmark.

Limitations

This work was limited to a review of the reports only. A small number of cases had obvious reporting issues; for example, it is impossible to have more positive lymph nodes than total lymph nodes. Ideally, the reporting framework should not allow logically inconsistent diagnostic parameters such as the one above. Likewise, it is not possible to have a positive lymph node and a pN0 nodal stage.

This analysis cannot provide insight into the criteria pathologists use in practice to count lymph nodes. Another aspect that could be studied is the experience level of the gross prosector and the pathologist; these factors were not captured.

Patient factors, tissue acquisition factors and laboratory processing factors were not considered in the analysis; however, as cases, in our environment, are usually assigned to the pathologist based on an irregular call schedule: the cases assignment is likely close to a random assignment. The minimum number of cases (>50 for each pathologist) and near random case assignment reduces the likelihood that confounders not considered significantly alter the conclusions.

The submission of more tissue by the pathologist (when less than 12 lymph nodes were retrieved) is suspected to be a predictor of meeting the 12 lymph node target; this information was not captured.

Conclusion

The lymph node count in our institution varied significantly between pathologists, while the positive lymph node call rate had a good consistency. The variation in lymph node count could not be explained by the absence or presence of tumour deposits and likely depends on how individual pathologists count and whether they submit more tissue for examination. Standardized criteria for lymph node counting should be developed and could be aided by the use of synoptic reporting data.

Supporting information

S1 File. Description of data.
(DOCX)

S1 Data. Anonymized data set (complete).
(CSV)

S2 Data. Anonymized data set (trimmed).
(CSV)

S3 Data. All tables including supplemental tables.
(XLS)

S1 Fig.
(TIF)

S2 Fig.
(ZIP)

Acknowledgments

An earlier version of this work was present at the Annual Meeting of the *United States and Canadian Academy of Pathology* (2021).

Author Contributions

Conceptualization: Michael Bonert.

Data curation: Michael Bonert.

Formal analysis: Michael Bonert.

Methodology: Michael Bonert.

Project administration: Pierre Major.

Software: Michael Bonert.

Supervision: Ipshita Kak, Michael Bonert.

Validation: Michael Bonert.

Visualization: Michael Bonert.

Writing – original draft: Shivali Srivastava, Michael Bonert.

Writing – review & editing: Shivali Srivastava, Ipshita Kak, Pierre Major, Michael Bonert.

References

1. Hermanek P, Henson DE, Hutter RVP, et al. Appendix II. Colorectal carcinoma. UICC TNM supplement—a commentary on uniform use. Berlin: Springer-Verlag, 1993:69–71.
2. Orsenigo E, Gasparini G, Carlucci M. Clinicopathological Factors Influencing Lymph Node Yield in Colorectal Cancer: A Retrospective Study. Gastroenterol Res Pract. 2019;5197914. https://doi.org/10.1155/2019/5197914 PMID: 30804995; PMCID: PMC6362492.
3. Swanson RS, Compton CC, Stewart AK, Bland KI. The prognosis of T3N0 colon cancer is dependent on the number of lymph nodes examined. Ann Surg Oncol. 2003; 10(1):65–71. https://doi.org/10.1245/aso.2003.03.058 PMID: 12513963.
4. Kelder W, Inberg B, Schaapveld M, Karrenbeld A, Grond J, Wiggers T, et al. Impact of the number of histologically examined lymph nodes on prognosis in colon cancer: a population-based study in the Netherlands. Dis Colon Rectum. 2009; 52(2):260–7. https://doi.org/10.1007/DCR.0b013e3181979164 PMID: 19279421.
5. Le Voyer TE, Sigurdson ER, Hanlon AL, Mayer RJ, Macdonald JS. Colon cancer survival is associated with increasing number of lymph nodes analyzed: a secondary survey of intergroup trial INT-0089. J Clin Oncol. 2003; 21(15):2912–2919. https://doi.org/10.1200/JCO.2003.05.062 PMID: 12885809
6. Vather R, Sammour T, Kahokehr A, Connolly AB, Hill AG. Lymph node evaluation and long-term survival in Stage II and Stage III colon cancer: a national study. Ann Surg Oncol. 2010; 17(2):289–296.
7. Frederick L G, Page DL, Fleming ID, Fritz AG, Balch CM, Haller DG, et al. AJCC cancer staging manual [Internet]. 6th ed. Greene FL, Page DL, Fleming ID, Fritz AG, Balch CM, Haller DG, et al., editors. New York, NY: Springer; 2013. Available from: http://dx.doi.org/10.1007/978-1-4757-3656-4
8. Feinstein AR, Sosin DM, Wells CK. The Will Rogers Phenomenon. Stage migration and new diagnostic techniques as a source of misleading statistics for survival in cancer. New England Journal of Medicine. 1985 Jun 20; 312(25):1604–8. https://doi.org/10.1056/NEJM198506203122504 PMID: 4000199.

9. Leung AM, Scharf AW, Vu HN. Factors Affecting Number of Lymph Nodes Harvested in Colorectal Cancer. Journal of Surgical Research. 2011 Jun; 168(2):224–30. https://doi.org/10.1016/j.jss.2009.09.001 Epub 2009 Sep 25. PMID: 20036394

10. Sherbeck JP, Zhao L, Lieberman RW. High Variability in Lymph Node Counts Among an International Cohort of Pathologists: Questioning the Scientific Validity of Node Counts. J Natl Compr Canc Netw. 2018 Apr; 16(4):395–401. https://doi.org/10.6004/jnccn.2017.7064 PMID: 29632059.

11. Parkash V, Bifulco C, Feinn R, Concato J, Jain D. To count and how to count, that is the question: interobserver and intraobserver variability among pathologists in lymph node counting. Am J Clin Pathol. 2010 Jul; 134(1):42–9. https://doi.org/10.1309/AJCPO92DZMUCGEUF PMID: 20551265.

12. Lopez-Chavez A. In CRC, distinguishing tumor deposit from lymph node. CAP Today. 2018; 32(11):1, 12–13. Available from: https://www.captodayonline.com/in-crc-distinguishing-tumor-deposit-from-lymph-node/. Accessed April 10, 2023.

13. Rock JB, Washington MK, Adsay NV, Greenson JK, Montgomery EA, Robert ME, et al. Debating deposits: an interobserver variability study of lymph nodes and pericolonic tumor deposits in colonic adenocarcinoma. Arch Pathol Lab Med. 2014 May; 138(5):636–42. https://doi.org/10.5858/arpa.2013-0166-OA PMID: 23902577; PMCID: PMC3935980.

14. AJCC. Colon and Rectum Cancer Staging. In: AJCC Cancer Staging Manual, Eighth Edition. New York: Springer; 2017: 251–264. https://doi.org/10.1007/978-3-319-40618-3_18

15. Chen SL, Steele SR, Eberhardt J, Zhu K, Bilchik A, Stojadinovic A. Lymph Node Ratio as a Quality and Prognostic Indicator in Stage III Colon Cancer. Annals of Surgery. 2011 Jan; 253(1):82–7. https://doi.org/10.1097/SLA.0b013e3181ffa780 PMID: 21135690.

16. Nelson H, Sargent DJ, Wieand HS, Fleshman J, Anvari M, Stryker SJ, et al. A Comparison of Laparoscopically Assisted and Open Colectomy for Colon Cancer. New England Journal of Medicine. 2004 May 13; 350(20):2050–9. https://doi.org/10.1056/NEJMoa032651 PMID: 15141043.

17. Srigley JR, McGowan T, Maclean A, Raby M, Ross J, Kramer S, et al. Standardized synoptic cancer pathology reporting: a population-based approach. J Surg Oncol. 2009 Jun 15; 99(8):517–24. https://doi.org/10.1002/jso.21282 PMID: 19466743

18. Ong ML, Schofield JB. Assessment of lymph node involvement in colorectal cancer. World J Gastrointest Surg. 2016 Mar 27; 8(3):179–92. https://doi.org/10.4240/wjgs.v8.i3.179 PMID: 27022445; PMCID: PMC4807319.

19. Lester SC. Special issues in specimen processing/lymph nodes. In: Manual of Surgical Pathology. Elsevier—Health Science; 2022.

20. Yeh CC, Pan CF, Liu HW, Lin JC, Fang LH, Lee HS, et al. Using the Fat-Clearing Technique to Improve Lymph Node Retrieval in Colorectal Cancer. Int J Surg Pathol. 2021 Jun; 29(4):385–391. https://doi.org/10.1177/1066896920975501 PMID: 33243057.

21. Bonert M, Naqvi A, Rahman M, Marshall JK, Xenodemetropoulos T, Arora P, et al. Stability of diagnostic rate in a cohort of 38,813 colorectal polyp specimens and implications for histomorphology and statistical process control. Sci Rep. 2021 Aug 20; 11(1):16942. https://doi.org/10.1038/s41598-021-95862-2 PMID: 34417490; PMCID: PMC8379162.

22. Bonert M, Collins A, Xenodemetropoulos T, Dmetrichuk JM, Al-Haddad S, Major P, et al. Application of Next Generation Quality/Statistical Process Control and Expert-Led Case Review to Increase the Consistency of Diagnostic Rates in Precancerous Colorectal Polyps. Qual Manag Health Care. 2021 Jul-Sep 01; 30(3):176–183. https://doi.org/10.1097/QMH.0000000000000299 PMID: 33405466; PMCID: PMC8219089.

23. Marks JH, Valsdottir EB, Rather AA, Nweze IC, Newman DA, Chernick MR. Fewer than 12 lymph nodes can be expected in a surgical specimen after high-dose chemoradiation therapy for rectal cancer. Dis Colon Rectum. 2010 Jul; 53(7):1023–9. https://doi.org/10.1007/DCR.0b013e3181dadeb4 PMID: 20551754.

24. Hammer N. In CRC, Distinguishing Tumor Deposit from Lymph Node. CAP Today. [Internet]. Available from: https://www.captodayonline.com/in-crc-distinguishing-tumor-deposit-from-lymph-node/. Accessed May 28, 2023.

25. Ahuja AT, Ying M, Ho SY, Antonio G, Lee YP, King AD, et al. Ultrasound of malignant cervical lymph nodes. Cancer Imaging. 2008 Mar 25; 8(1):48–56. https://doi.org/10.1102/1470-7330.2008.0006 PMID: 18390388; PMCID: PMC2324368.
